# *Paracoccus kondratievae* produces poly(3‐hydroxybutyrate) under elevated temperature conditions

**DOI:** 10.1111/1758-2229.13260

**Published:** 2024-06-05

**Authors:** Radwa Moanis, Hannelore Geeraert, Niko Van den Brande, Ulrich Hennecke, Eveline Peeters

**Affiliations:** ^1^ Research Group of Microbiology, Department of Bioengineering Sciences Vrije Universiteit Brussel Brussels Belgium; ^2^ Faculty of Sciences, Botany and Microbiology Department Damanhour University Damanhour Egypt; ^3^ Research Group of Physical Chemistry and Polymer Science Vrije Universiteit Brussel Brussels Belgium; ^4^ Research Group of Organic Chemistry, Department of Chemistry and Department of Bioengineering Sciences Vrije Universiteit Brussel Brussels Belgium

## Abstract

As part of ongoing efforts to discover novel polyhydroxyalkanoate‐producing bacterial species, we embarked on characterizing the thermotolerant species, *Paracoccus kondratievae*, for biopolymer synthesis. Using traditional chemical and thermal characterization techniques, we found that *P. kondratievae* accumulates poly(3‐hydroxybutyrate) (PHB), reaching up to 46.8% of the cell's dry weight after a 24‐h incubation at 42°C. Although *P. kondratievae* is phylogenetically related to the prototypical polyhydroxyalkanoate producer, *Paracoccus denitrificans*, we observed significant differences in the PHB production dynamics between these two *Paracoccus* species. Notably, *P. kondratievae* can grow and produce PHB at elevated temperatures ranging from 42 to 47°C. Furthermore, *P. kondratievae* reaches its peak PHB content during the early stationary growth phase, specifically after 24 h of growth in a flask culture. This is then followed by a decline in the later stages of the stationary growth phase. The depolymerization observed in this growth phase is facilitated by the abundant presence of the PhaZ depolymerase enzyme associated with PHB granules. We observed the highest PHB levels when the cells were cultivated in a medium with glycerol as the sole carbon source and a carbon‐to‐nitrogen ratio of 10. Finally, we found that PHB production is induced as an osmotic stress response, similar to other polyhydroxyalkanoate‐producing species.

## Graphical abstract

*Paracoccus kondratievae* is a thermotolerant bacterium that is demonstrated to be a PHB producer in this study. It accumulates PHB upon growth on several carbon sources with different carbon/nitrogen ratios and as a response to osmotic stress conditions. At elevated temperatures, growth kinetics as well as the kinetics of PHA accumulation were found to be different as compared to the known mesophilic PHA producer *Paracococcus denitrificans*.

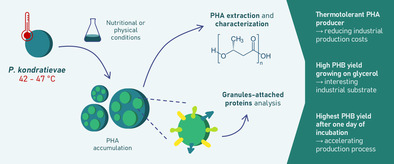

## INTRODUCTION

Microbially produced polyhydroxyalkanoates (PHAs) have gained industrial interest as these polyesters constitute a sustainably produced, non‐toxic and biodegradable alternative to petroleum‐based plastics (Kovalcik et al., 2019). PHAs are biosynthesized by numerous prokaryotic microorganisms during unbalanced nutritional conditions, especially in case of a high carbon‐to‐nitrogen ratio and are accumulated in intracellular granules as a means of carbon and energy storage (Jendrossek, 2009; Shah & Kumar, 2021). In addition, PHA synthesis is stimulated in response to specific stress conditions, such as high salinity, and has been proposed to play a role in cellular protection during such stress conditions (Obruča et al., 2022).

A large phylogenetic diversity of bacterial and archaeal species have a native PHA‐producing capability (Kim & Rhee, 1999; Kourilova et al., 2020; Mohandas et al., 2018; Tu et al., 2022; Yamane et al., 1996). Within the gram‐negative genus *Paracoccus*, several species were characterized as PHA producers, including *Paracoccus pantotrophus* (Ucisik‐Akkaya et al., 2009), *Paracoccus homeinsis* (Szacherska et al., 2022) and *Paracoccus denitrificans* (Yamane et al., 1996). The latter species has been extensively studied for its PHA‐producing capability. It is a mesophilic facultative methylotrophic species that produces high levels of poly(3‐hydroxybutyrate) (PHB). During growth on glycerol as a carbon source, *P. denitrificans* was found to produce 72% of PHB in cell dry weight (CDW) (Kalaiyezhini & Ramachandran, 2015). When cultivated on methanol and *n*‐amyl alcohols, the species is also capable of producing the copolymer poly(3‐hydroxybutyrate‐*co*‐3‐hydroxyvalerate) (PHBV) (Ueda et al., 1992). *P. denitrificans* is also considered prototypical given that the regulation of PHA synthesis has been elaborately studied in the species, which led to the identification of a dedicated regulator named PhaR (Gao et al., 2001; Maehara et al., 2002). PhaR is a transcriptional repressor of the phasin‐encoding gene *phaP* and has unique properties, as it binds both DNA and PHA granules (Yamada et al., 2007).

Despite extensive knowledge on PHA synthesis in *P. denitrificans* and other model PHA producers, the industrial valorization of microbial PHA production is still limited. A major bottleneck that impedes widespread commercialization is the relative high production cost: as compared to petroleum‐based plastics, the cost of PHA production is often 4–5 times higher (D'Souza, 2018). A possible solution is the use of thermophilic or thermotolerant strains, which would lower PHA production costs due to a higher cultivation temperature implying lowered cooling energy and minimized sterility precautions (Chavan et al., 2021; Obruča et al., 2018). In previous studies, various thermophilic strains have been explored for PHA production, including *Caldimonas thermodepolymerans* (Kourilova et al., 2020; Obruča et al., 2022), *Aneurinibacillus* spp. (Pernicova et al., 2020), *Chelatococcus thermostellatus* (Ibrahim et al., 2016), *Tepidimonas taiwanensis* (Kourilova et al., 2021) and *Cupriavidus cauae* (An et al., 2023).

Here, we turn our focus to a species that belongs to the *Paracoccus* genus and that has a thermotolerant lifestyle. *Paracoccus kondratievae*, originally isolated from the maize rhizosphere, was reported to grow optimally at temperatures between 38 and 42°C (Doronina et al., 2002). It grows aerobically and can utilize a large diversity of organic substrates, besides being a facultative chemolithotroph and methylotroph (Doronina et al., 2002). The strain *P. kondratievae* BJQ0001 has been shown to possess the capacity to enzymatically degrade phthalate esters, which are plasticizers typically liberated from plastics into the environment and induce severe health risks for humans and animals (Xu et al., 2020). Another strain, *P. kondratievae* CRT2, was found to be proficient in generating substantial amounts of carotenoid pigments during its growth on pretreated lignocellulosic waste (Pyter et al., 2022). These observations, together with the demonstration of fast growth on low‐cost substrates such as methanol and molasses and the currently ongoing genetic tool development, underscore the large biotechnological potential of *P. kondratievae* in the context of a biobased and circular economy (Maj et al., 2020).

Given its phylogenetic relatedness to the well‐known PHA producer *P. denitrificans* (Yamane et al., 1996), it could be hypothesized that *P. kondratievae* is a potentially promising novel industrial host for a competitive PHA production process at higher temperatures. However, to our knowledge, *P. kondratievae* has not been investigated yet for its potential to accumulate PHA. In this work, we set out to investigate the PHA production ability of *P. kondratievae* in comparison to the well‐known PHA producer *P. denitrificans*. The structural and thermal characteristics of the extracted biopolymers were studied, and moreover, granule‐associated proteins were identified with a mass spectrometry approach. Fluorescence‐based observations of PHA accumulation enabled the study of the process in different growth phases, temperature, medium compositions, with varying carbon/nitrogen (C/N) ratios and elevated NaCl concentrations. As such, this study reveals similarities but also unique differences in PHA synthesis characteristics in the thermotolerant *P. kondratievae* with respect to the model PHA‐producing bacterium *P. denitrificans*.

## EXPERIMENTAL PROCEDURES

### Bioinformatic analysis

The annotated genome sequence of chromosomes 1 and 2 of *P. kondratievae* strain BJQ0001 was retrieved from the National Center of Biotechnology Information (NCBI) database (GenBank accession numbers CP045072.1 and CP045073.1, respectively). Using nucleotide sequences of PHA‐related genes from *P. denitrificans* PD1222, the basic local alignment search tool ‘tblastn’ was used to search for putative *pha* genes in *P. kondratievae* BJQ0001. Multiple sequence alignments of protein sequences were constructed using the Sequence Manipulation Suite (https://www.bioinformatics.org/sms2/). Genomic environments and gene syntenies in different *Paracoccus* species were explored and visualized using the SyntTax tool (Oberto, 2013).

For prediction of putative promoter elements in intergenic regions in the *pha* gene clusters, 200‐bp sequences of the upstream regions of open reading frames were analysed with the BPROM algorithm (Solovyev & Salamov, 2011).

### Bacterial strains and growth conditions

The strain *P. kondratievae* NCIMB13773 was obtained from the National Collections of Industrial, Food and Marine Bacteria (NCIMB), UK, while *P. denitrificans* DSM413 was obtained from the German Collection of Microorganisms and Cell Cultures (DSMZ). *P. kondratievae* cells were initially cultivated at 42°C in tryptic soy broth (TSB) medium (Schau, 1986; Smith & Dell, 1990), while *P. denitrificans* was cultivated at 30°C in lysogeny broth (LB) (Sambrook & Russell, 2001). After overnight incubation, precultures were used as inoculum with a concentration of 3% to reach an initial optical density at 600 nm (OD_600_) of 0.3 ± 0.05, for cultivation in mineral salt medium (MSM) (Pantazaki et al., 2003). This medium had the following composition: 15 g L^−1^ sodium gluconate unless indicated otherwise, 1 g L^−1^ NH_4_Cl, 10 g L^−1^ NaCl, 1.2 g L^−1^ KH_2_PO_4_, 0.26 g L^−1^ K_2_HPO_4_·3H_2_O, 19.9 mg L^−1^ CaCl_2_·2H_2_O, 123 mg L^−1^ MgSO_4_·7H_2_O and 6 mL L^−1^ of a mineral solution containing 1.96 g L^−1^ H_3_PO_4_, 56 mg L^−1^ FeSO_4_·7H_2_O, 29 mg L^−1^ ZnSO_4_·7H_2_O, 16.7 mg L^−1^ MnSO_4_·H_2_O, 2.5 mg L^−1^ CuSO_4_·5H_2_O, 3 mg L^−1^ Co(NO_3_)_2_·6H_2_O and 6 mg L^−1^ H_3_BO_3_. The pH of the medium was set to 7.0 ± 0.2 with NaOH. In experiments in which the carbon source was altered, sodium gluconate was replaced by 15 g L^−1^ xylose, galactose or glucose or by 15 mL L^−1^ glycerol or waste frying oil. In carbon‐to‐nitrogen (C/N) ratio experiments, the carbon source was present at different concentrations, while the nitrogen source NH_4_Cl was maintained constant at 1 g L^−1^. In experiments in which NaCl concentration was altered, *P. kondratievae* was cultivated in TSB and *P. denitrificans* in LB. Media were sterilized by autoclaving except for glucose, galactose and xylose stock solutions, which were filter‐sterilized. Cells were cultivated in 100‐mL shaking‐flasks with 30 mL culture volumes for fluorescence measurements and in 250‐mL shaking‐flasks with 100 mL culture volumes for PHA extraction. Growth was monitored by measuring OD_600_ using a spectrophotometer. Incubation was performed at 180 rpm and at 30°C for *P. denitrificans* and at 42°C for *P. kondratievae*, unless otherwise specified.

For the determination of growth parameters, OD_600_ values were plotted, linearly fitted and the exponential growth phase was determined manually based on plots of ln OD_600_ versus time (h). The specific growth rate (*μ*) was determined as the slope of these linear fits, and the doubling time (*t*_d_) was determined as ln(2)/*μ* (Baes et al., 2020).

### Nile Red staining of cells

To detect the presence of intracellular lipid‐like materials, the Nile Red staining technique was used (Zuriani et al., 2013). In an initial approach, fluorescence microscopy was used for visualization (Salgaonkar et al., 2013). To this end, 100 μL of culture was smeared on a glass slide, heat‐fixed and washed with distilled water, followed by the addition of a 0.01% Nile Red solution in DMSO. After an incubation of 20 min, excess stain was drained and stained cells were washed with distilled water, followed by air‐drying and addition of a cover slip. Next, the sample was visualized using a Nikon eclipse Ti2 microscope using the TRITC filter set with an excitation wavelength of 550 nm and an emission wavelength of 603.5 nm.

For plate reader detection, cells from 1 mL aliquots of various cultures were collected via centrifugation at 12,000*g* for 5 min. The subsequent pellet was reconstituted in 1 mL of distilled water, followed by the addition of 40 μL of a Nile Red solution with a concentration of 80 μg mL^−1^, dissolved in DMSO. The stained sample was allowed to incubate at room temperature for 30 min before being subjected to another centrifugation step for 5 min at 12,000*g*. Following this, the supernatant was removed and the pellet was reconstituted in 1 mL of distilled water. Finally, 150 μL of the reconstituted sample was placed into a well of a 96‐well plate. Nile Red fluorescence was gauged using a plate reader (Synergy, Biotek) with an excitation wavelength of 535 nm and an emission wavelength of 605 nm, employing a gain of 70. In the same assay, OD_600_ was measured. To determine the relative quantity of intracellular lipid‐like components concerning cell density, the fluorescence signal was normalized by the measured OD_600_ (FL/OD).

### PHA extraction

PHA extraction was performed as previously described (Mozejko‐Ciesielska et al., 2017). Briefly, *P. kondratievae* cells were cultivated during 24 h in 100 mL‐volume cultures and centrifuged at 12,108*g*, 4°C for 10 min. The pellets were then lyophilized and afterwards, they were incubated with 20 mL chloroform in coned 50‐mL Falcon centrifugation tubes, while shaking at 180 rpm and 50°C for 3 h. Subsequently, the mixture was filtered through No. 1 Whatman filter paper and the filtrate was left to dry at room temperature. PHA content was calculated as a ratio of the weight of extracted PHA relative to the CDW of the lyophilized cells (Johnston et al., 2018):

$$\mathrm{PHA}\;\text{content}\left(\%\right)=\begin{array}{l} \left(\text{weight of extracted}\;\mathrm{PHA}/\text{weight of}\;\mathrm{CDW}\right)\\ \times 100\%. \end{array}$$

### Fourier transform infrared spectroscopy

Fourier transform infrared (FTIR) spectroscopy was applied for further chemical analysis. Infrared (IR) spectra were obtained using a Nicolet 6700 FTIR spectrophotometer (Thermo Fisher Scientific). It was operated in a single bounce attenuated total refractance (ATR) mode using the Smart iTR accessory. A diamond plate with a 42° angle of incidence was used. Using this system, 32 scans (resolution 4 cm^−1^) were taken between 600 and 4000 cm^−1^ to form the final IR spectra. FTIR analysis was also applied to poly(3‐hydroxybutyrate‐*co*‐3‐hydroxyvalerate) (PHBV, 2% 3‐HV) as a reference (Sigma Aldrich). Spectra were cut between 1900 and 700 cm^−1^ and scaled to a maximum absorbance of 1 within this region.

### Gas chromatography coupled to mass spectrometry

To prepare the samples for gas chromatography coupled to electron impact mass spectrometry (GC–MS), 2 mg of extracted PHAs was resuspended in 600 μL of 10% sulfuric acid in methanol, followed by incubation at 100°C for 4 h, enabling conversion to β‐hydroxycarboxylic acid methyl esters. After allowing the methanol solution to cool to room temperature, 1 mL of distilled water and two times 0.5 mL of dichloromethane were added to the sample, which was shaken vigorously for 1 min. After phase separation, the organic layers were collected and solvent was evaporated. Next, *N*,*O*‐Bis(trimethylsilyl)acetamide was added and the mixture was heated to 60°C for 1 h. This step was performed to obtain the silylated equivalents. Finally, 10 μL of the mixture was diluted with ethyl acetate (1 mL), of which 1 μL was subjected to GC–MS analysis (Brandl et al., 1988). Resulting methyl esters were analysed on a Shimadzu GC‐MS QP5050A system running with helium as a carrier gas and equipped with a split/splitless injector and an Agilent HP‐5MS column (column length: 30 m; column diameter: 0.25 mm; film thickness: 0.25 μm). The program used was 2 min hold time at 50°C, followed by 15°C min^−1^ to 300°C.

### Nuclear magnetic resonance spectroscopy

As a means to analyse the extracted polymers with proton nuclear magnetic resonance (^1^H NMR) spectroscopy, about 3 mg was solubilized in 0.7 mL deuterated chloroform (CDCl_3_). ^1^H NMR spectra were recorded on a Bruker Avance 250 spectrometer at 250 MHz using a standard pulse sequence. ^1^H NMR chemical shifts (*δ*) are reported in ppm relative to TMS and referenced to the residual solvent signal (CDCl_3_: 7.26 ppm).

### Differential scanning calorimetry

The thermal properties of the extracted polymers, as well as of PHBV (2% 3‐HV) (Goodfellow) as a standard, were analysed by differential scanning calorimetry (DSC), which was performed using a Discovery DSC (TA Instruments) equipped with a refrigerated cooling system under a nitrogen flow (50 mL min^−1^). For each DSC measurement, approximately 3 mg of sample was placed in Tzero pans with Tzero hermetic lids (TA Instruments). Each sample was first heated (20 K min^−1^) to 200°C and then maintained for 1 min, cooled (20 K min^−1^) to −50°C and maintained for 1 min and finally increased again (20 K min^−1^) to 200°C and maintained for 1 min. Melting temperature (*T*_m_) determination was based on the maximum of the endothermic peak during the second heating cycle.

### Thermogravimetric analysis

The thermal stability of the extracted PHA samples was characterized by performing thermogravimetric analysis (TGA). The same protocol was applied to a solvent‐casted sheet of commercial PHB (Goodfellow) as a standard. For this purpose, 2.5–3.5 mg of PHA was placed on platinum pans, loaded into the TGA Q5000 machine (TA Instruments), and heated up to 650°C (20 K min^−1^) under nitrogen flow (25 mL min^−1^). The onset decomposition temperature (*T*_d_) was determined using the TA Universal Analysis tool as the temperature where the starting‐mass baseline intersects with the tangent to the TGA curve at its steepest point.

### Isolation of PHA granules

PHA granules were extracted from a 300 mL culture that was cultivated for 24 h on MSM with sodium gluconate as a carbon source, using glycerol gradient ultracentrifugation (Li et al., 2019). Cells were harvested through centrifugation for 20 mins at 4500*g* and 4°C. Subsequently, pellets were washed and resuspended in a 50 mM phosphate buffer (pH 7.5), which was then subjected to sonication for 45 mins. A volume of 3.5 mL of cell lysates was loaded onto a glycerol gradient comprising 44% and 88% (v/v) glycerol in a phosphate buffer and subjected to ultracentrifugation at 235,000*g* and 4°C for 3.0 h using a Beckman Coulter (Optima L‐90K) ultracentrifuge equipped with a 45 Ti Beckman rotor. PHA granules were distinctly observed as a white layer above the 88% glycerol stratum. They were extracted through pipetting, followed by washing with 10 volumes of a 50 mM phosphate buffer (pH 7.5) and centrifugation at 235,000*g* for 60 mins at 4°C. The collected granules formed a white pellet. This pellet was either directly subjected to liquid chromatography and mass spectrometry analysis or resuspended in 5 mL of phosphate buffer for sodium‐dodecyl sulfate polyacrylamide gel electrophoresis (SDS‐PAGE) analysis. FTIR analysis was also performed to confirm the presence of PHA in the sample.

### Shotgun liquid chromatography with tandem mass spectrometry

Liquid chromatography with tandem mass spectrometry (LC–MS/MS) was performed at the VIB Proteomics Core. To prepare the PHB granule sample for analysis, 200 μL of buffer (10% SDS and 100 mM triethylammonium bicarbonate [TEAB], pH 8.5) was added to the pellet. The SDS concentration was adjusted to 5% with 50 mM TEAB to a final volume of 400 μL. The sample was point‐sonicated three times for 10 s and put on ice in between the sonication steps. After 15 mins of centrifugation at maximum speed, proteins were reduced and alkylated by addition of 10 mM Tris(2‐carboxyethyl)phosphine (TCEP) and 40 mM chloroacetamide, followed by incubation for 10 mins at 95°C at 750 rpm in the dark. Phosphoric acid was added to a final concentration of 1.2% and subsequently the sample was diluted to sevenfold with binding buffer containing 90% methanol in 100 mM TEAB, pH 7.55. Next, it was loaded on a 96‐well S‐TrapTM plate (Protifi) in parts of 400 μL, placed on top of a deep‐well plate and centrifuged for 2 mins at 1500*g* at 20°C. After protein binding, the S‐trapTM plate was washed three times by adding 200 μL of binding buffer and centrifugating for 2 mins at 1500*g* at 20°C. A new deep‐well receiver plate was placed below the 96‐well S‐TrapTM plate and 125 μL 50 mM TEAB containing 1 μg of trypsin was added for digestion overnight at 37°C. Using centrifugation for 2 mins at 1500*g*, peptides were eluted thrice, first with 80 μL of 50 mM TEAB, then with 80 μL of 0.2% formic acid (FA) in water and finally with 80 μL of 0.2% FA in water/acetonitrile (ACN) (50/50, v/v). Eluted peptides were dried completely by vacuum centrifugation and redissolved in 0.1% TFA in water/ACN (98:2, v/v) for an additional purification step on Omix C18 tips (Agilent). Finally, purified peptides were dried completely by vacuum drying and redissolved in 20 μL loading solvent A (0.1% trifluoroacetic acid in water/acetonitrile (ACN) (98:2, v/v)).

Next, 1 μL was injected for LC–MS/MS analysis on an Ultimate 3000 RSLCnano system in‐line connected to a Q Exactive HF Biopharma mass spectrometer (Thermo). Trapping was performed at 20 μL min^−1^ for 2 mins in loading solvent A on a 5 mm trapping column (Thermo Scientific, 300 μm internal diameter, 5 μm beads). Peptides were separated on a 1.9 μm C18, 75 μm inner diameter column (made in‐house, packed in needle, beads from Dr. Maisch, Germany) and kept at a constant temperature of 45°C. Peptides were eluted by a non‐linear gradient starting at 1% MS solvent B reaching 26% MS solvent B (0.1% FA in water/acetonitrile (2:8, v/v)) in 75 min, 44% MS solvent B (0.1% FA in water/acetonitrile (2:8, v/v)) in 95 mins, 56% MS solvent B in 100 mins followed by a 5‐min wash at 56% MS solvent B and re‐equilibration with MS solvent A (0.1% FA in water).

The mass spectrometer was operated in a data‐dependent mode, automatically switching between MS and MS/MS acquisition for the 16 most abundant ion peaks per MS spectrum. Full‐scan MS spectra (375–1500 *m*/*z*) were acquired at a resolution of 60,000 in the Orbitrap analyser after accumulation to a target value of 3,000,000. The 16 most intense ions above a threshold value of 15,000 were isolated with a width of 1.5 m/z for fragmentation at a normalized collision energy of 28% after filling the trap at a target value of 100,000 for maximum of 80 ms. MS/MS spectra (200–2000 *m*/*z*) were acquired at a resolution of 15,000 in the Orbitrap analyser.

### Proteomic data analysis

Analysis of the mass spectrometry data was performed in MaxQuant (version 2.1.3.0) with mainly default search settings, including a false discovery rate set at 1% on peptide spectrum matches, on both peptide and protein levels. Spectra were searched against the protein sequence database of *Paracoccus_kondratieva*_txid135740. The mass tolerance for precursor and fragment ions was set to 4.5 and 20 ppm, respectively, during the main search. Enzyme specificity was set as C‐terminal for arginine and lysine, also allowing cleavage at proline bonds with a maximum of two missed cleavages. Carbamidomethylation of cysteine residues was set as a fixed modification. Variable modifications were set to oxidation of methionine residues and formylation of protein N‐termini. Protein abundances were obtained by the iBAQ algorithm.

## RESULTS

### The *P. kondratievae* genome harbours conserved PHA synthesis gene clusters

**FIGURE 1:**
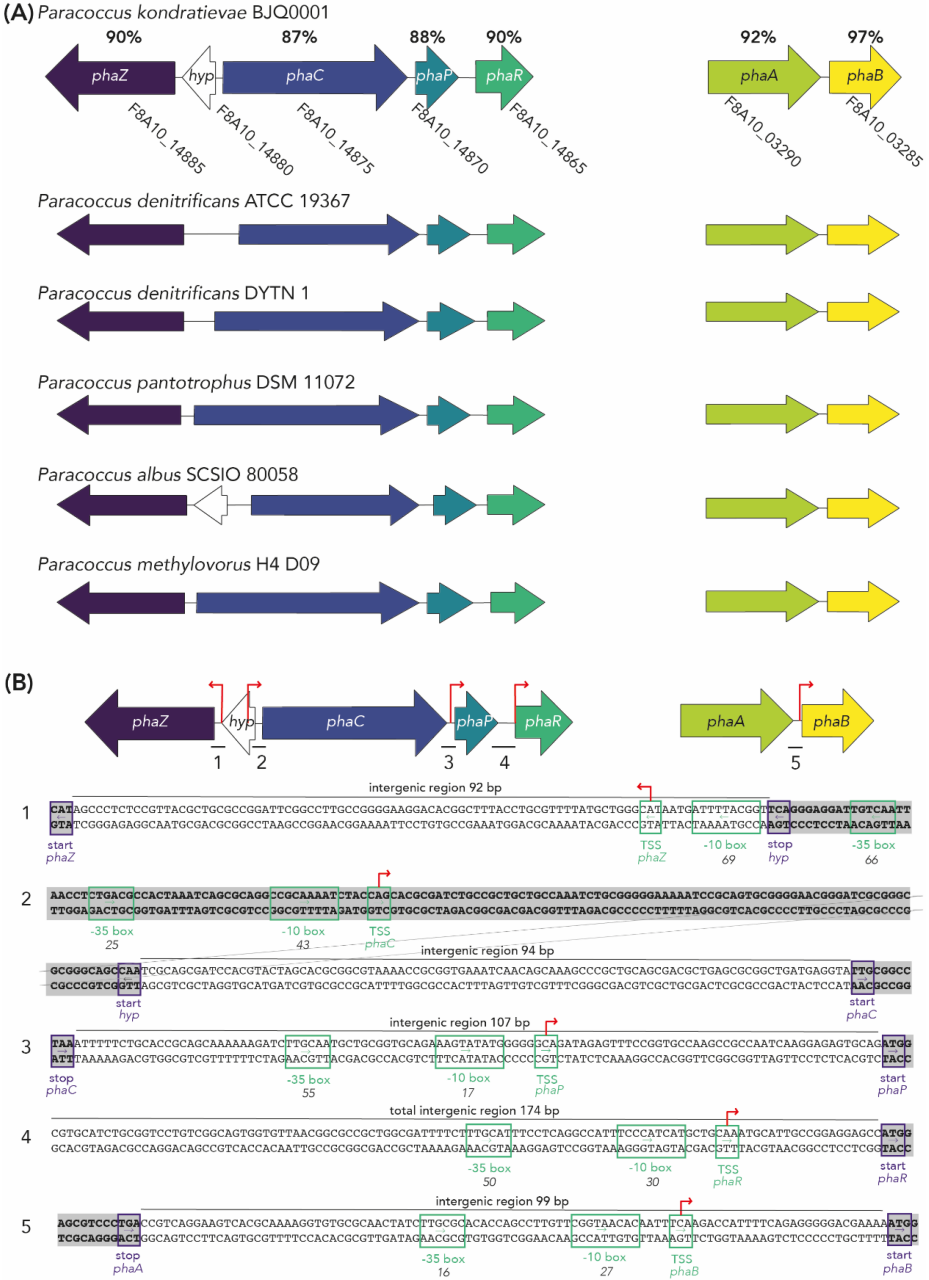
Genotypic indication for PHA production in *P. kondratievae*. (A) Schematic representation of the genetic organization of PHA‐related genes in *P. kondratievae* BJQ0001, including gene numbers and amino acid sequence identities with homologues in *P. denitrificans* PD1222. Below, gene syntenies of the corresponding PHA gene clusters in selected *Paracococcus* strains are schematically represented, based on an analysis in SyntTax (Oberto, 2013). (B) In silico analysis of the transcriptional structure of the PHA gene clusters in *P. kondratievae*. Nucleotide sequences of intergenic regions are depicted, with indication of translational start and stop codons (boxed in purple), putative −10 and −35 promoter elements (boxed in green) and putative transcription start sites (TSSs) (region boxed in green, TSS indicated with a red arrow). Coding regions are shaded in grey, while the length of the intergenic region is mentioned. For the *phaP‐phaR* intergenic region, only the part immediately upstream of *phaR* is shown, while the length of the total intergenic region is mentioned. The BPROM algorithm was used for predicting −10 and −35 boxes, with the prediction scores mentioned below the boxes in italics. TSSs were predicted manually based on their spacing with the −10 box.

To initiate the investigation into the PHA accumulation ability of *P. kondratievae*, a genotypic analysis was performed (Figure 1). The annotated genome sequence of *P. kondratievae* BJQ0001 was retrieved from NCBI, where it was reported to be organized on two different chromosomes. Based on PHA‐related gene sequences in *P. denitrificans*, homologous genes were retrieved in the *P. kondratievae* genome sequence, in a similar genomic organization (Figure 1A). PHA genes are organized in two distinct gene clusters, with one cluster on Chromosome 1 harbouring *phaA*‐ and *phaB*‐encoding genes and a second cluster on Chromosome 2 harbouring *phaP*, *phaR*, *phaC* and *phaZ* arranged in a divergent orientation.

This gene synteny and organization in two distinct gene clusters were also found to be conserved in a wider array of *Paracoccus* species, including *P. pantotrophus*, *Paracoccus albus* and *Paracoccus methylovorus* (Figure 1A). In this gene synteny analysis, variability was observed in the predicted length of *phaC*, encoding the key enzyme PHA synthase (PhaC) and in the length of the intergenic region between the divergently encoded *phaC* and *phaZ*, even for different *P. denitrificans* strains. In addition, for some genomes, including that of *P. kondratievae* BJQ0001, a small gene encoding a hypothetical protein was predicted within this intergenic region (Figure 1A). Amino acid sequence identities between *P. kondratievae* and *P. denitrificans* PHA‐related proteins range between 87% and 97%, with functional homology for the key enzyme PhaC being corroborated by conservation of the catalytic triad, consisting of a cysteine within a lipase‐like box ([GS‐X‐C‐X‐[GA]‐G]), an aspartate and a histidine (Figure S1).

Regardless of the clustering of PHA genes on the genome, it was unclear whether or not some of the genes are expressed in transcriptional units. The relatively large intergenic regions separating each of the genes suggest the latter, a hypothesis that was further investigated by performing an in silico prediction of promoter elements (Figure 1B). Indeed, for each of the genes, with the exception of *phaA* and the small gene encoding a hypothetical protein, promoter elements were predicted, indicating independent transcriptional expression and a lack of operonic organization.

### P. kondratievae *synthesizes PHB*

**FIGURE 2:**
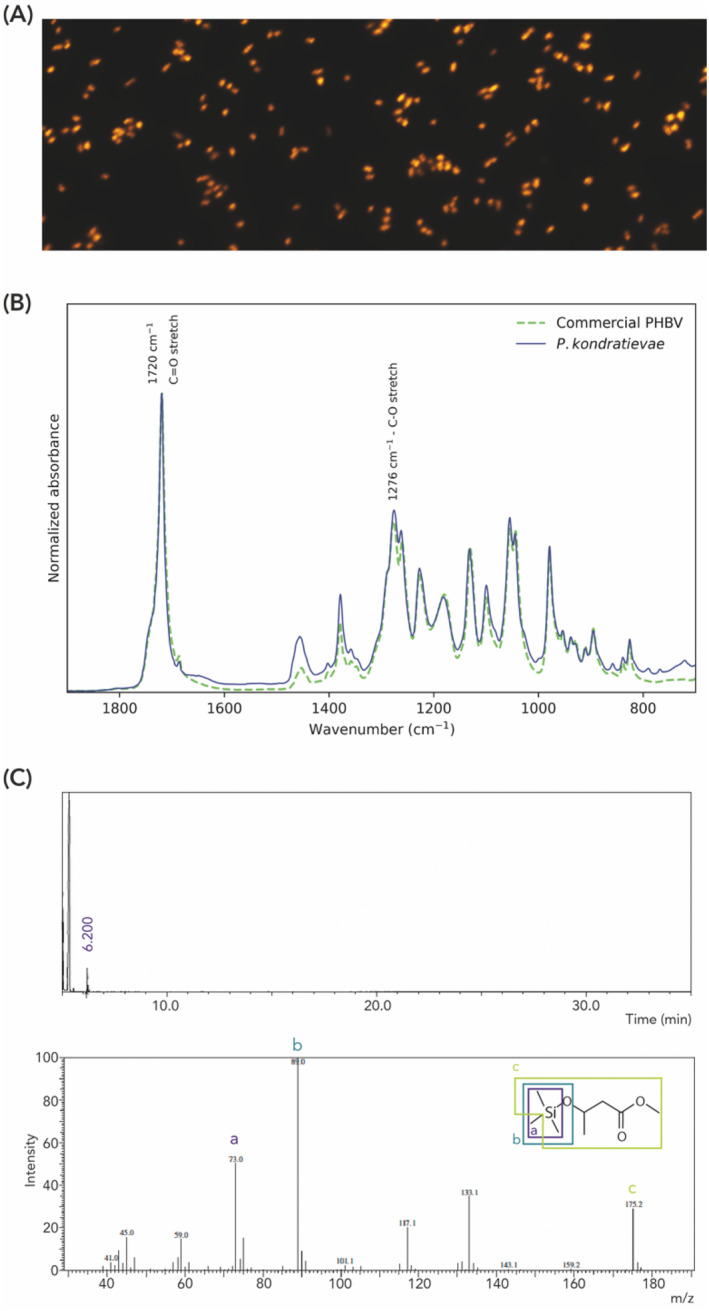
Chemical and thermal analysis of PHA extracted from *P. kondratievae*. (A) Nile Red stained cells of *P. kondratievae* NCIMB13773 cells visualized with fluorescence microscopy. (B) FTIR spectrum of the extracted PHA sample as compared to that of commercial PHBV as a reference. Characteristic PHA peaks are indicated by their respective wavenumber. (C) GC–MS analysis of the extracted polymer. Top: GC chromatogram of the extracted PHA sample. The *x*‐axis represents the retention time and the *y*‐axis depicts a quantitative presentation of the number of molecules with the same retention time. The peak with a retention time of 6.200 min, indicated in purple letter type, is hypothesized to be PHB as this is the characteristic retention time. Bottom: MS spectrum of the main peak with retention time 6.2 min; *x*‐axis: relative mass of the charged cation compound (*m*/*z*); *y*‐axis: relative intensity of the occurrence of cations formed during fragmentation at the start of MS. At the top‐right corner, a 3‐(trimethylsilyl)‐methyl ester derived of a 3HB monomer is displayed. The most important peaks on the graph are indicated with a letter that correspond to a specific fragment of the 3‐(trimethylsilyl)‐methyl ester.

Regardless of the genetic presence of a PHA synthesis machinery in the *P. kondratievae* genome similar to that in *P. denitrificans*, its activity needs to be confirmed experimentally. To this end, *P. kondratievae* NCIMB13773 was cultivated in MSM with sodium gluconate as a sole carbon source and subjected to Nile Red staining and microscopy imaging (Figure 2A), which is a classical and fast methodology to visualize intracellular inclusions of hydrophobic compounds such as PHA granules. In correspondence with the presence of PHA‐containing granules, fluorescent dots were observed within the *P. kondratievae* cells (Figure 2A).

To confirm that the detected intracellular fluorescence indeed represents PHA and to further characterize the chemical and thermal properties of the biopolymers, a chloroform‐based PHA extraction procedure was performed on lyophilized cells, yielding a white substance characteristic for PHA. The obtained CDW was found to have a PHA content of 27.6%, corresponding to a PHA concentration of 0.5 g L^−1^ of culture. Different chemical analysis methods were performed to examine the nature of the extracted polymer (Figure 2B,C, Supplementary Text S1, Figures S2–S4). The FTIR spectrum was found to show a very high correspondence to that of the reference sample, PHBV, which confirmed that the extracted polymer is indeed a PHA polymer (Figure 2B). In these FTIR spectra, peaks located at 1719 cm^−1^ (C=O stretch), 1453 cm^−1^ (CH_2_ aliphatic stretching), 1379 cm^−1^ (CH_3_ vibration), 1277 and 1228 cm^−1^ (C—O stretching) are indicative for PHA polymers.

Next, ^1^H NMR and GC–MS were performed to further analyse the monomeric composition of the extracted PHA (Figure 2C and Figure S2). The NMR spectrum displayed typical peaks and chemical shifts of a PHB homopolymer (Figure S2), which was further confirmed by GC–MS, revealing a single main peak with a retention time of 6.2 mins (Figure 2C). Mass spectrometry analysis revealed indeed a typical fingerprint of the 3‐(trimethylsilyl)‐methyl ester of 3‐hydroxybutyrate, the silylated derivative of the monomer of PHB. Thermal characteristics of the polymer were subsequently investigated using DSC and TGA, confirming the sole presence of PHB (Supplementary Text S1 and Figures S3 and S4). Altogether, these results demonstrate that not only genetic make‐up but also chemical composition is identical for both *P. denitrificans* (Kalaiyezhini & Ramachandran, 2015; Maehara et al., 2001) and *P. kondratievae*: they both synthesize PHB homopolymers.

### PHB production as a function of cultivation temperature and time

**FIGURE 3:**
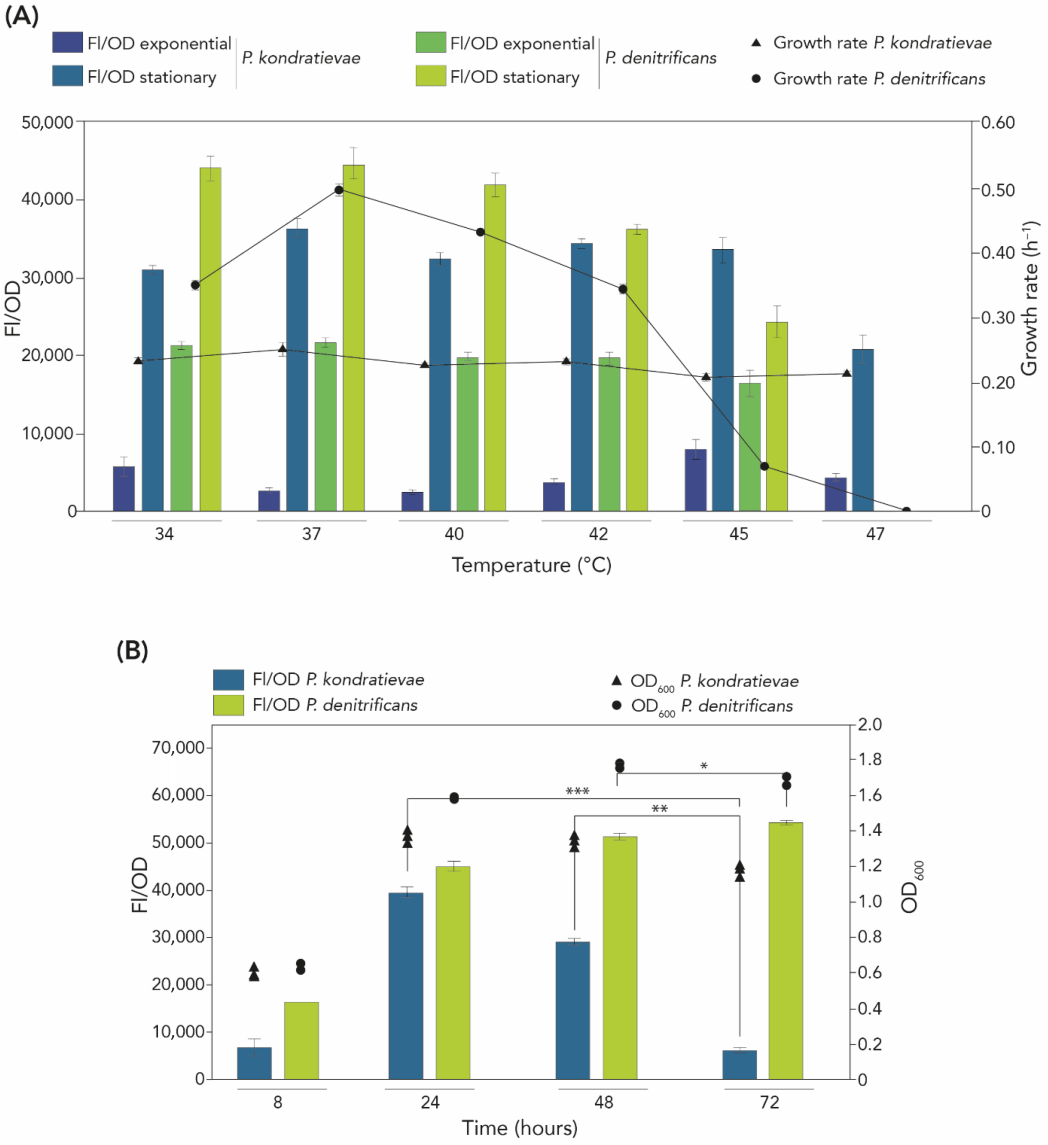
Temperature and time dependence of growth and PHB production in *P. kondratievae* and *P. denitrificans*. (A) FL/OD values, OD_600_ values and growth rates were determined for flask cultures of *P. kondratievae* NCIMB13773 and *P. denitrificans* DSM413. (A) Fluorescence and OD_600_ values were measured at time points 10 h (corresponding to exponential growth phase) and 24 h (corresponding to stationary growth phase). Growth rates were calculated for the growth curve segments representing exponential growth. (B) FL/OD and OD_600_ values measured for *P. denitrificans* and *P. kondratievae* flask cultures during stationary growth phase at specified time points. Cultivation was performed at 42°C for *P. kondratievae* NCIMB13773 and at 30°C for *P. denitrificans* DSM413. Statistical significance was calculated using a paired *t*‐test (*p < 0.05; **p < 0.01; ***p < 0.001).

In light of a previously observed linear correlation between Nile Red staining‐based fluorescence levels and PHA content (Bordel et al., 2021), we employed this approach to follow PHB accumulation throughout the growth process (Figure 3).

Given the premise that *P. kondratievae* has thermotolerant growth characteristics and might be capable of producing PHB at a higher temperature than *P. denitrificans*, we investigated the effect of temperature on growth and PHB synthesis for both species. Fluorescence/OD_600_ (FL/OD) values were determined as a semi‐quantitative measure of PHB amounts at different time points in the growth process (10 and 24 h, representing exponential and early stationary growth phase) and at different temperatures (Figure 3A). These experiments demonstrated that *P. kondratievae* has a markedly different thermal growth profile as compared to *P. denitrificans*. For all temperatures tested within the range of 34–47°C, the growth rate of *P. kondratievae* remained very similar, averaging 0.23 ± 0.01 h^−1^. In contrast, growth of *P. denitrificans* displayed a bell curve temperature dependence with a maximal growth rate of 0.50 ± 0.01 h^−1^ at 37°C, while growth was significantly impaired at 45°C and completely ceased at 47°C. In terms of PHB synthesis, the experiments revealed that the difference in PHB concentration between the exponential and stationary growth phases is larger for *P. kondratievae* than for *P. denitrificans*, with the former surpassing the latter in the early stationary growth phase at 45°C. Moreover, in contrast to *P. denitrificans*, *P. kondratievae* was found to be synthesizing PHB at 47°C in the early stationary growth phase at a similar growth rate as compared to lower temperatures (Figure 3A).

Next, we turned our focus to the stationary growth phase, given that this appeared to be the most interesting phase with regards to PHB yield based on FL/OD measurements. This demonstrated that while intracellular PHA accumulation in *P. denitrificans* increased over time during the stationary growth phase, *P. kondratievae* exhibited an inverse trend, displaying the highest FL/OD value at 24 h, followed by a gradual decrease at later time points (Figure 3B). These differences in FL/OD levels were corroborated by the observation that OD_600_ values decreased for *P. kondratievae* upon comparing the 24‐ and 72‐h time points, while this was not the case for *P. denitrificans*. Overall, these results demonstrated that PHB production and degradation appear to be more tightly regulated in response to growth dynamics in *P. kondratievae* as compared to *P. denitrificans* and that the highest concentrations are obtained in the early stationary growth phase. In addition, a declining OD_600_ indicates that *P. kondratievae* is subjected to cell lysis during stationary growth.

### Identification of PHA‐associated proteins in the granules

To further explore the composition of PHA granules formed in *P. kondratievae* with a focus on the associated proteins, we performed a granule isolation procedure based on glycerol gradient ultracentrifugation (Li et al., 2019). Cells were harvested during the early stationary growth phase, after 24 h of cultivation, which corresponds to the growth phase with the highest levels of PHB accumulation (Figure 3B), as is the case for other bacterial species (McCool et al., 1996; Tufail et al., 2017). FTIR analysis confirmed the presence of PHAs in the extracted sample (Figure S5). Next, LC–MS/MS analysis of the preparation enabled identification of proteins within the preparation, which represent granule‐attached proteins (GAPs) (Table 1, Supplementary Dataset S1). A comprehensive proteome analysis resulted in the detection of 1751 proteins, including PhaA, PhaB, PhaC, PhaP, PhaZ and PhaR.

**TABLE 1:** Proteins detected in PHA granules purified from *P. kondratievae* NCIMB13773, as revealed by LC–MS/MS analysis.

Identified proteins	Proteins IDs	MW (kDa)	iBAQ value (×10^3^)
Phasin (PhaP)	**QFQ88734.1**	**16.03**	**22,582,000**
Porin	QFQ87926.1	35.01	7,855,500
Polyhydroxyalkanoate depolymerase (PhaZ)	**QFQ88736.1**	**49.81**	**2,168,300**
Transcription termination factor Rho	QFQ85964.1	47.34	1,889,100
DUF883 family protein	QFQ89400.1	15.88	1,504,300
SCO family protein	QFQ88460.1	22.39	1,363,900
30S ribosomal protein S21	QFQ86162.1	8.012	1,341,300
50S ribosomal protein L16	QFQ87559.1	15.71	1,273,600
Imidazoleglycerol‐phosphate dehydratase HisB	QFQ87222.1	21.51	1,174,200
Antibiotic ABC transporter	QFQ88599.1	11.64	1,092,100
Outer membrane beta‐barrel protein	QFQ88203.1	21.70	887,470
OmpA family protein	QFQ89328.1	22.82	866,480
Polyhydroxyalkanoate synthesis repressor (PhaR)	**QFQ88733.1**	**22.11**	**708,950**
30S ribosomal protein S14	QFQ87553.1	11.85	707,670
DUF481 domain‐containing protein	QFQ88102.1	34.00	687,740
NADP‐specific glutamate dehydrogenase	QFQ87381.1	49.53	660,410
Pyruvate dehydrogenase complex dihydrolipoamide acetyltransferase	QFQ87365.1	45.25	649,330
30S ribosomal protein S3	QFQ87560.1	27.02	575,200
Ribosomal protein S5	QFQ87549.1	20.49	554,660
30S ribosomal protein S12	QFQ87570.1	14.18	541,970
Hypothetical protein F8A10_07470	QFQ87276.1	14.90	536,710
F0F1 ATP synthase subunit alpha	QFQ88388.1	55.10	513,210
50S ribosomal protein L35	QFQ86156.1	7.362	508,700
DUF4167 domain‐containing protein	QFQ86838.1	32.37	501,130
H‐NS histone family protein	QFQ89384.1	11.89	497,560
30S ribosomal protein S11	QFQ87543.1	13.87	486,790
H‐NS histone family protein	QFQ87944.1	11.54	478,640
hypothetical protein GCM10017635_31430	GLK65666.1	9.55	477,300
30S ribosomal protein S15	QFQ87924.1	10.22	472,490
F0F1 ATP synthase subunit beta	QFQ88386.1	50.23	469,250
50S ribosomal protein L2	QFQ87563.1	30.37	432,190
Pyruvate dehydrogenase complex E1 component subunit beta	QFQ87364.1	48.65	428,700
TonB‐dependent receptor (plasmid)	QFQ89633.1	74.79	428,640
30S ribosomal protein S4	QFQ87189.1	23.72	418,970
30S ribosomal protein S16	QFQ87374.1	13.78	406,800
30S ribosomal protein S13	QFQ87544.1	13.63	401,830
Hypothetical protein F8A10_10450	QFQ87816.1	11.77	394,230
l,d‐Transpeptidase family protein	QFQ88064.1	25.53	383,860
Pyruvate dehydrogenase (acetyl‐transferring) E1 component subunit alpha	QFQ87363.1	37.37	361,310
Preprotein translocase subunit YajC	QFQ88097.1	11.71	351,030
META domain‐containing protein	QFQ89229.1	14.27	349,530
50S ribosomal protein L24	QFQ87555.1	10.89	347,210
Hypothetical protein F8A10_02200	QFQ86342.1	11.43	345,680
Hypothetical protein F8A10_11580	QFQ88154.1	7.222	340,800
Bifunctional glutamate N‐acetyltransferase/amino‐acid acetyltransferase ArgJ	QFQ87713.1	48.34	335,960
50S ribosomal protein L19	QFQ87371.1	14.12	310,590
TonB‐dependent receptor	QFQ86530.1	78.18	308,570
Hypothetical protein F8A10_11575	QFQ88153.1	7.663	299,130
TonB‐dependent receptor	QFQ87726.1	72.74	292,680
Manganese/iron ABC transporter ATP‐binding protein	QFQ86936.1	31.82	286,060

*Note*: Abundance is indicated by the iBAQ value, which corresponds to the sum of all peptide intensities divided by the number of observable peptides of a protein. The 50 most abundant proteins are shown here, and the entire dataset is presented in Supplementary Dataset S1. Proteins implicated in PHA synthesis are indicated in bold.

The most abundant protein was PhaP, which is a hydrophobic phasin and typically represents the major constituent of the outer surface of PHA granules. In addition, a porin (QFQ87926.1) and the depolymerase PhaZ were also retrieved with high abundance (Table 1). While PhaA, PhaB and PhaC were not retrieved within the top‐50 most abundant proteins, they were detected with lower abundances (iBAQ values of 12,805 × 10^3^, 9233 × 10^3^ and 120,380 × 10^3^, respectively) (Supplementary Dataset S1). These results confirm the expression of the PHA machinery that was originally identified to be encoded in the genome (Figure 1A) and its association with PHB. The relative high abundance of PhaZ within the granules might be attributed to the PHB synthesis/degradation dynamics that are specific for *P. kondratievae* (Figure 3B). Indeed, quickly after reaching the stationary growth phase in a flask culture, PHB concentrations decreased, indicating an abundant depolymerization activity attributed to the PhaZ depolymerase enzyme. This observation is in contrast to PHB cycling dynamics in *P. denitrificans*.

### Effect of carbon‐to‐nitrogen ratio on PHB production

**FIGURE 4:**
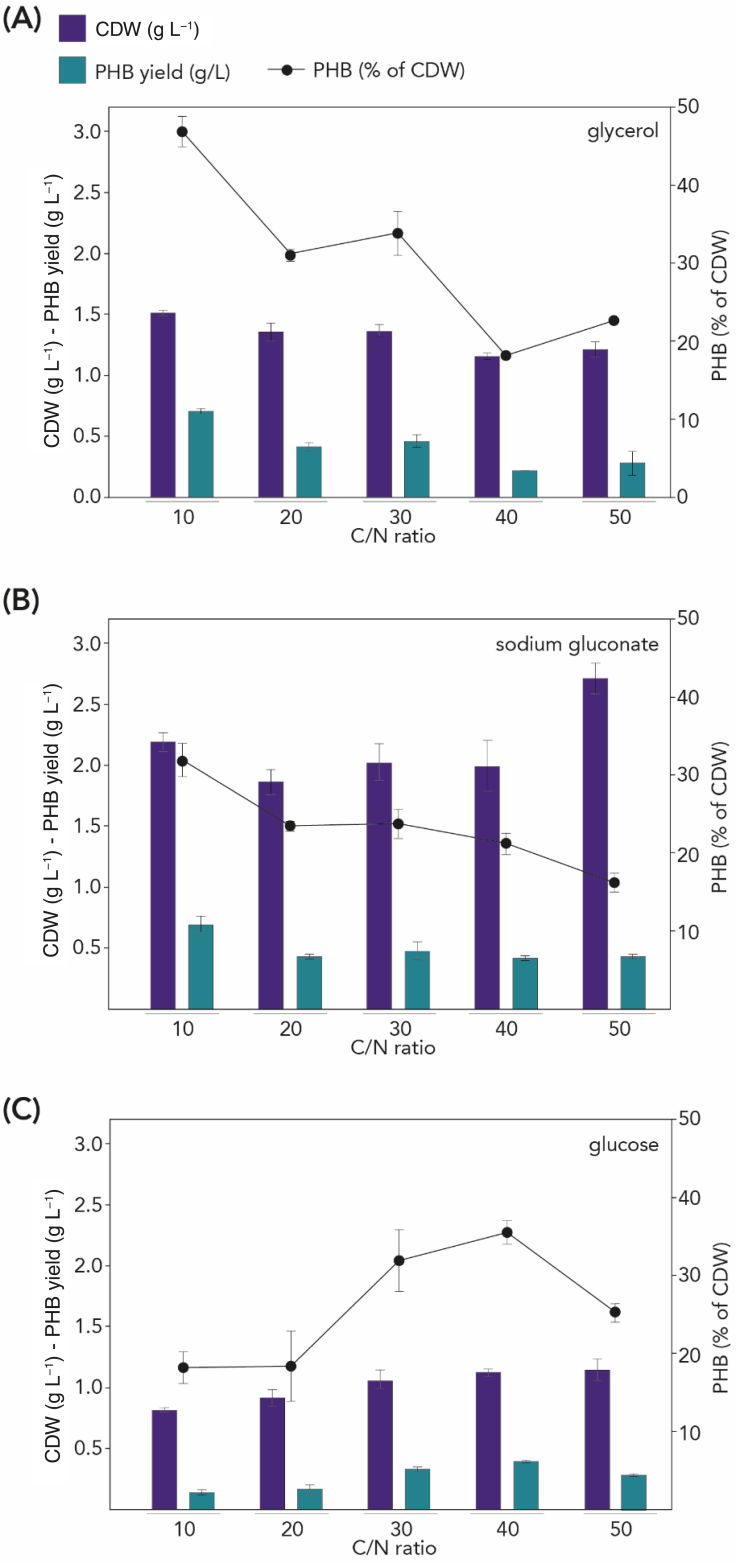
Effect of C/N ratio on PHB production by *P. kondratievae* NCIMB13773. Cultivation was performed in MSM with glycerol (A), sodium gluconate (B) or glucose (C) as a sole carbon source and cells were harvested after 24 h of growth. Corresponding data are presented in Table S1 and results of statistical tests in Supplementary Dataset S2.

Given the metabolic versatility of *Paracoccus* species, we screened the growth of *P. kondratievae* on other carbon sources than sodium gluconate (Figure 4). While xylose, galactose and waste frying oil did not support growth of the strain, glycerol and glucose did. Upon supplementing the medium with alternative carbon sources instead of sodium gluconate, similar trends were observed for the PHB accumulation profiles of the strains (Figure S6A,B). As it is well established that an imbalance in the C/N ratio in the medium induces PHA production (Zhou et al., 2022), we investigated the effect of altering the C/N ratio on PHB accumulation after 24 h of growth in *P. kondratievae* for different carbon sources (Figure 4A–C, Table S1 and Supplementary Dataset S2). Overall, it was observed that the use of sodium gluconate as a carbon source resulted in the highest CDW values for all C/N ratios tested, indicating that cells grow best in this condition. In contrast, glucose resulted in the lowest biomass amounts.

To assess PHB yields, the biopolymers were extracted from cells for quantification. It was observed that the use of glycerol as a sole carbon source resulted in the highest PHB content of 46.8%, at a C/N ratio of 10 (Figure 4A). Also, upon cultivating the cells on sodium gluconate, the lowest C/N ratio (10) resulted in the highest PHB content (32%) (Figure 4B). At higher C/N ratios, the PHB content decreased both upon cultivation on glycerol and on sodium gluconate, although in the latter case, the amount of biomass increased and was maximal at the highest C/N ratio of 50 (2.7 g L^−1^), thereby mitigating the decrease in obtained PHB concentration (g L^−1^) as the PHB content was lowest (16.3%). In contrast, upon cultivating the cells in a medium with glucose as a sole carbon source, an increase in PHB content was observed that was directly proportional with the increase in C/N ratio until reaching a ratio of 40, at which it was 35.6% (Figure 4C). At a C/N ratio of 50, the PHA content decreased again to 25.3%.

### Effect of high salt concentrations on PHB production

**FIGURE 5:**
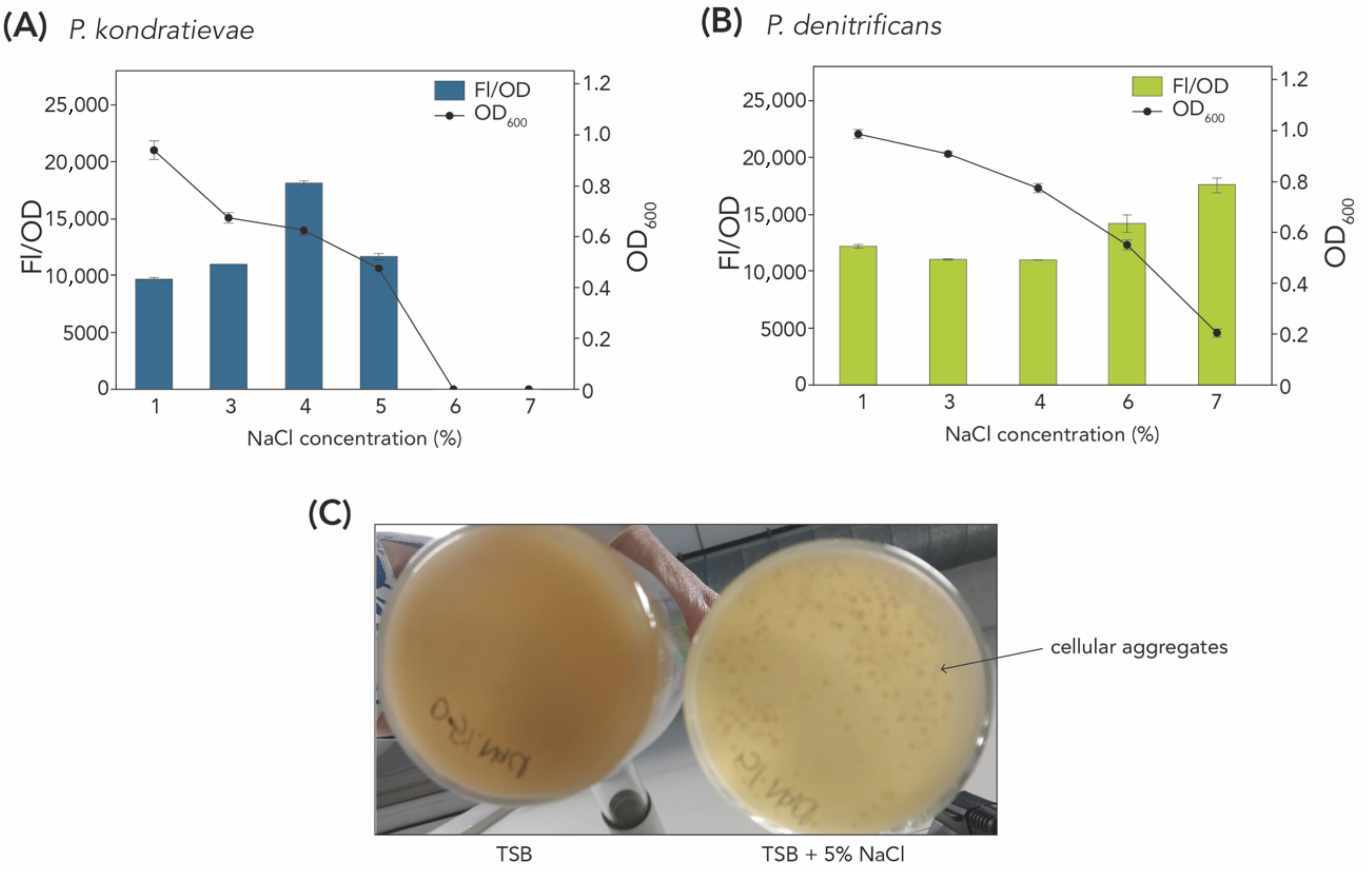
Effect of NaCl concentration on growth and PHA accumulation. This experiment was performed for (A) *P. kondratievae* cultivated in TSB medium and (B) *P. denitrificans* cultivated in LB medium. Samples were analysed after 72 h. A statistical analysis could not be performed for these data because duplicate measurements were performed. (C) Bottom‐view picture taken of cultures of *P. kondratievae* cultivated on TSB medium (left) and TSB + 5% NaCl (5%), with indication of cell aggregates for the latter.

For many PHA‐producing bacterial species, osmotic stress is known to induce PHA accumulation, an observation that could be relevant for optimization of yield and productivity. Therefore, the effect of osmotic stress on growth and PHA accumulation was examined for both *P. kondratievae* and *P. denitrificans* by augmenting the salinity concentration in the nutrient‐rich growth medium (Figure 5). Increased NaCl concentrations impacted growth in an inversely proportional manner until it ceased entirely. It was demonstrated that *P. denitrificans* survives in NaCl concentrations up to 7%, whereas *P. kondratievae* only displays growth up to 5% NaCl. Conversely, as osmotic stress increased, FL/OD measurements, which are a proxy for PHB accumulation, increased linearly up to 4% NaCl for *P. kondratievae* (Figure 5A). These findings corresponded with those for *P. denitrificans*, for which PHA accumulation increased with increasing salinity concentrations up to 7% NaCl (Figure 5B). Notably, the elevation of salinity concentration in the medium for *P. kondratievae* led to the formation of cell aggregates (Figure 5C and Figure S7).

## DISCUSSION

In this work, we provided experimental evidence that *P. kondratievae* is capable of synthesizing PHB at temperatures ranging from 34 to 47°C. At 42°C and upon growth on glycerol, a PHB yield was obtained of 46.8% of CDW. As such, this species can be added to the list of thermotolerant PHA producers. Previously reported strains include *Cupriavidus* sp. CB15, capable of producing up to 75% PHA of CDW upon cultivation on glycerol at 45°C (Yootoum et al., 2023), *Bacillus thermoamylovorans*, capable of producing PHAs up to 63% of CDW upon cultivation on sodium octanoate at 45°C (Choonut et al., 2020), and *Aneurinibacillus* sp., which produces PHB up to 55% of CDW upon cultivation on glycerol at 45°C (Pernicova et al., 2020).

Most genetically encoded PHA proteins were identified as being associated with PHA granules, underscoring their involvement in PHA synthesis (Table 1). Phasins were found to be the most dominant GAPs, which is not surprising given their major structural role in forming the PHA granules (Mezzina & Pettinari, 2016). A specific porin protein was abundantly present as well, in accordance with observations for PHB granules from *P. denitrificans* (Maehara et al., 1999) and other bacterial species such as *Cupriavidus necator* (Jendrossek & Pfeiffer, 2014). In addition, many other proteins were detected as well, most with functional roles that are very different from PHA metabolism. It can be hypothesized that most of these proteins are false‐positive GAPs, explained by a nonspecific binding to the hydrophobic polymers after cell rupture during the extraction process, as found in other studies as well (Jendrossek & Pfeiffer, 2014; Liebergesell et al., 1992).

In agreement with the phylogenetically related *P. denitrificans*, *P. kondratievae* was shown to synthesize pure PHB. However, when comparing the dynamics of PHB synthesis in the time course of growth in a flask culture, notable differences were observed between both species. First of all, although *P. denitrificans* was shown to display a higher growth rate and PHB concentration than *P. kondratievae* at ambient temperatures, *P. kondratievae* was capable of growing and synthesizing PHB at elevated temperatures (45–47°C) at which the growth of *P. denitrificans* was strongly or entirely impaired (Figure 3A). For *P. kondratievae*, the highest level of PHB content was observed after 24 h of cultivation, followed by a fast decline upon progressing in the stationary growth phase (Figure 3B). In contrast, PHA content kept increasing in *P. denitrificans* and reached its highest level after 72 h. It was previously observed that PHB accumulation is higher in the stationary phase than the exponential growth phase in *P. denitrificans* (Kalaiyezhini & Ramachandran, 2015). A continued increase in PHB content during stationary phase growth might be explained by a strong flux of cellular Acetyl‐CoA towards PHB synthesis (Bordel et al., 2021). Not only synthesis, but also depolymerization contributes to the total PHA content. Similarly, in *P. kondratievae*, depolymerization activity increases in the early stationary phase, thereby causing a decrease in PHB content (Figure 3). This is in agreement with the observation of a high abundance of the PhaZ depolymerase enzyme in extracted granules (Table 1). This phenomenon can be explained by a cellular need of degrading PHB polymers into monomers so that they can be used as a carbon and energy source (Ong et al., 2017). It should also be noted that growth behaviour such as cell density and cell lysis during the stationary phase was also different upon comparing *P. denitrificans* with *P. kondratievae*.

Cultivation of *P. kondratievae* on MSM with glycerol as a sole carbon source with a C/N ratio of 10 resulted in a PHA content of 47% and a PHA concentration of 0.7 g L^−1^ (Figure 4). Similarly, glycerol as a sole carbon source also results in the highest PHA content, albeit with higher values up to 72% CDW and with a C/N ratio of 21.4 (mol mol^−1^) in *P. denitrificans* (Kalaiyezhini & Ramachandran, 2015). PHA levels varied with altered C/N ratios in different ways for both strains. However, for *P. kondratievae* PHA accumulation decreased at a C/N ratio higher than 40 for all carbon sources (Figure 4). This can be explained by an inhibition effect caused by high concentrations of carbon compounds (Sánchez Valencia et al., 2021). It was observed that PHA accumulation decreased by increasing the C/N ratio to a certain extent, which differed from one carbon source to another. Similar trends were observed in other studies, including upon the use of a dynamic mixed microbial consortium (Zhou et al., 2022).

The response to increased osmolarity was also different for the two *Paracoccus* species. While the apparent PHB content kept on increasing up to 7% NaCl for *P. denitrificans*, it reached a maximum at 4% NaCl and decreased thereafter for *P. kondratievae*, which exhibited a higher sensitivity to increasing salt concentrations (Figure 5). Indeed, at high salt concentrations, *P. kondratievae* displayed cellular aggregation, which has been described before in other microbial species such as *Planococcus halocryophilus* as an adaptation strategy to high salinity (Heinz et al., 2019). Nevertheless, for both *Paracoccus* strains, it was evident that by increasing the NaCl concentration in the medium, PHB synthesis is stimulated. This underscores the previous observations that in non‐halophilic bacteria, the formation of PHB granules is part of a stress response to high salinity (Obruča et al., 2017). It is hypothesized that the intracellular presence of granules aids in protecting the cytoplasmic membrane and reducing plasmolysis.

## AUTHOR CONTRIBUTIONS

**Radwa Moanis:** Conceptualization (equal); formal analysis (equal); investigation (lead); methodology (equal); writing – original draft (lead). **Hannelore Geeraert:** Formal analysis (equal); methodology (equal); writing – review and editing (equal). **Niko Van den Brande:** Methodology (equal); writing – review and editing (equal). **Ulrich Hennecke:** Methodology (equal); writing – review and editing (equal). **Eveline Peeters:** Conceptualization (equal); resources (lead); writing – review and editing (equal).

## CONFLICT OF INTEREST STATEMENT

The authors decalre no conflicts of interest.

## Supporting information


**Data S1.** Supporting Information.

**Data S2.** Supporting Information.

## Data Availability

All data are provided in the results and Supplementary Information of this paper.
