# Supplementary material for: Paracoccus kondratievae produces poly(3‐hydroxybutyrate) under elevated temperature conditions
Source: Environ Microbiol Rep. 2024 Jun 5;16(3):e13260. doi: 10.1111/1758-2229.13260 (PMC11150862; doi:10.1111/1758-2229.13260)
Supplement: Supplementary file 1 — Data S1. Supporting Information. [file EMI4-16-e13260-s001.pdf]

Supplementary Information Moanis *et al.*

Supplementary Text

Supplementary Text S1. Additional chemical and thermal characterization of extracted polyhydroxyalkanoates

Thermal characteristics of the polymer were subsequently investigated using DSC (**Supplementary Figure S2**) and TGA (**Supplementary Figure S3**), which operates through a programmed temperature regimen comprising consecutive heating and cooling cycles. Within these cycles, a semi-crystalline polymer undergoes distinct thermal transitions, including glass transition, exothermic crystallization and endothermic melting, which are influenced by several factors as the polymer's composition and molecular weight (Carrasco *et al.*, 2006). The DSC thermogram of the extracted sample from *P. kondratievae* revealed a melting temperature (T_m) of 167°C, consistent with established thermal properties of pure PHB homopolymers (Misra *et al.*, 2006). Due to the relatively high crystallinity acquired by the PHB polymer during cooling, the glass transition temperature (T_g) (associated with the amorphous phase) is challenging to detect. TGA analysis was applied to both a commercially available PHB and to the PHB sample extracted from *P. kondratievae* (**Supplementary Figure S3**). TGA curves exhibited a bimodal decomposition pattern, with onset decomposition temperatures (T_ds) of approximately 232°C and 227°C for PHB from *P. kondratievae* and from a commercial origin, respectively. The similarity of both T_d values underscores their equivalent thermal stability.

Supplementary Figures

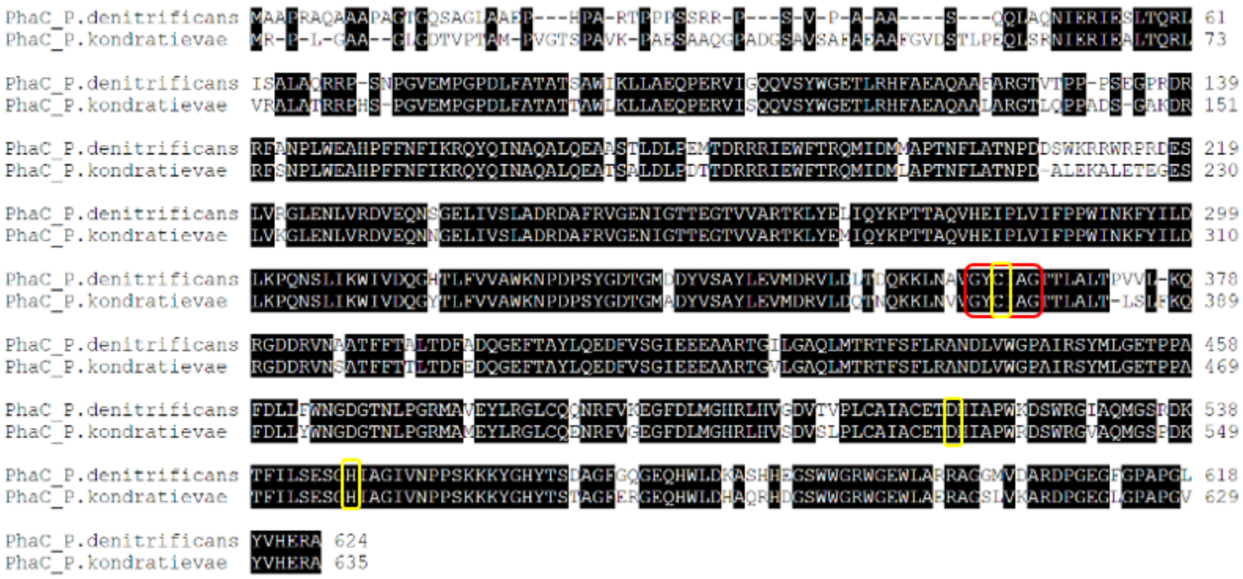

Supplementary Figure S1. Pairwise sequence alignment of PhaC from *P. denitrificans* and *P. kondratievae*. Identical residues are shaded in black, while the conserved lipase-like box is boxed in red and the catalytic residues are boxed in yellow.

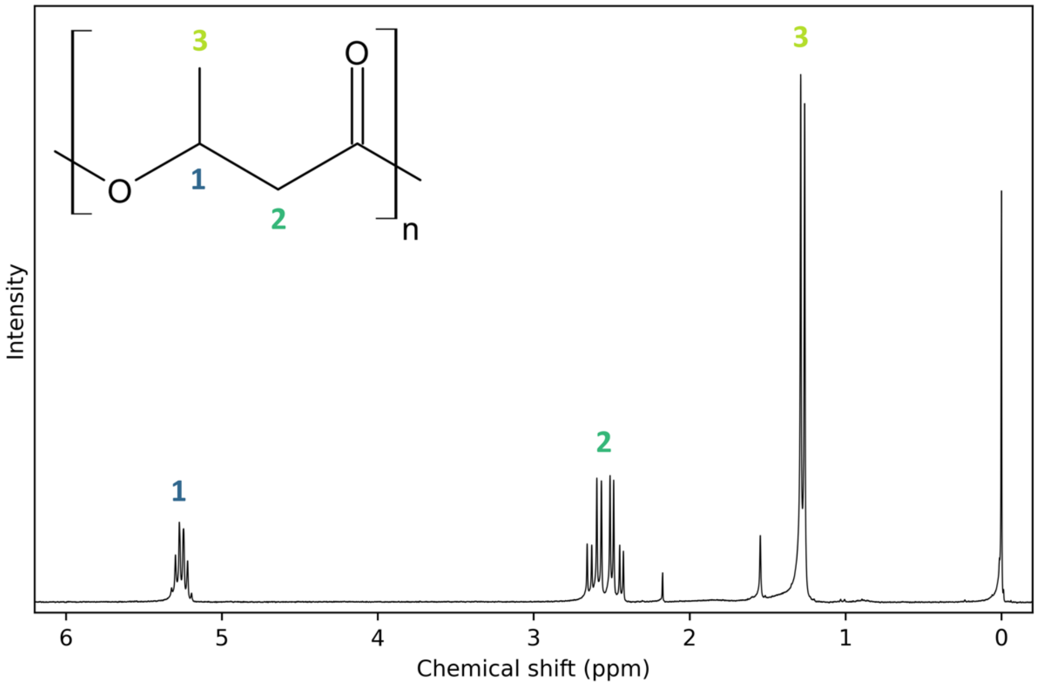

Supplementary Figure S2. Chemical analysis of a PHA sample extracted from *P. kondratievae* cells cultivated on MSM medium with sodium gluconate as a sole carbon source with NMR. ^{1}H NMR spectrum of the extracted PHA, with the Y-axis representing signal intensity and the X-axis indicating proton chemical shift for distinct peaks.

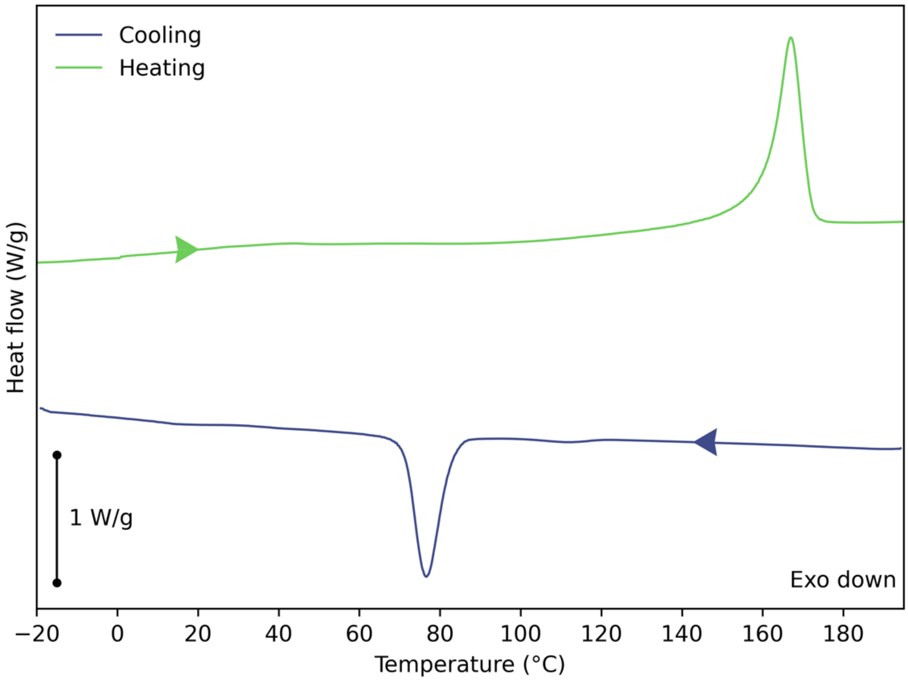

Supplementary Figure S3. DSC analysis of PHB extracted from *P. kondratievae*. DSC thermogram of the PHA sample, illustrating the cooling step (20 K/min to -50°C; blue) and the subsequent second heating step (20 K/min to 200°C; green). The x-axis represents temperature T (°C), while the y-axis represents normalized heat flow Q (W/g).

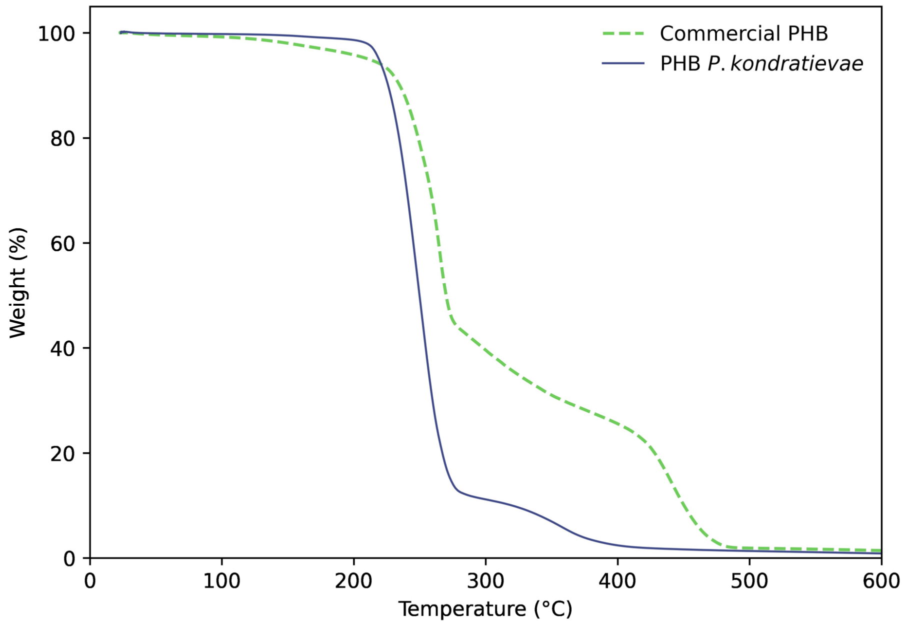

Supplementary Figure S4. TGA analysis of PHB extracted from *P. kondratievae* compared to commercial PHB. X-axis: temperature (°C); y-axis: weight loss (%).

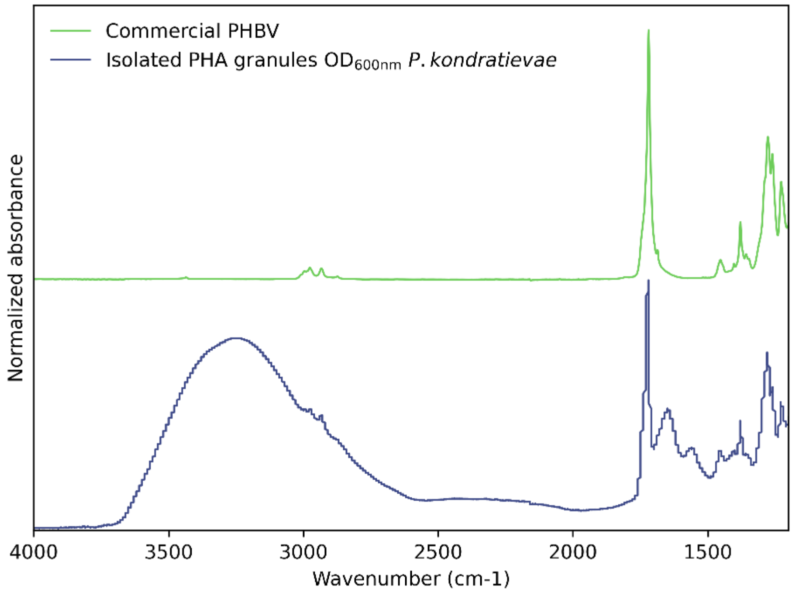

Supplementary Figure S5. FTIR spectrum of the extracted PHA granules. Commercial PHBV was analyzed as a reference.

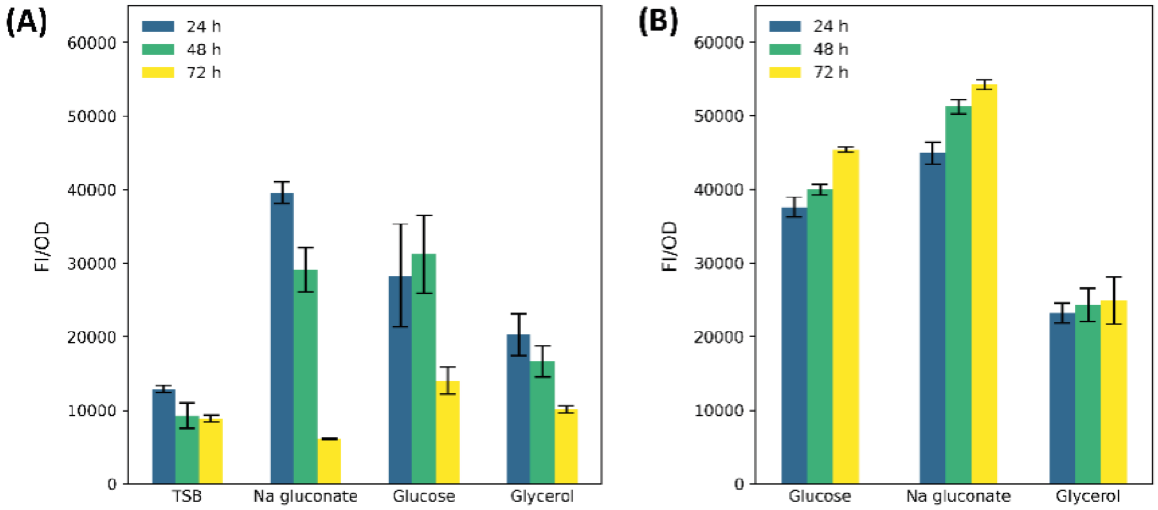

Supplementary Figure S6. FL/OD upon growth in different medium compositions. (A) *P. kondratievae* cells cultivated on rich medium (TSB) and on minimal MSM medium with different carbon sources, as indicated. Experiments were performed in triplicate. **(B)** *P. denitrificans* cells cultivated on minimal MSM medium with different carbon sources, as indicated. Experiments were performed in duplicate.

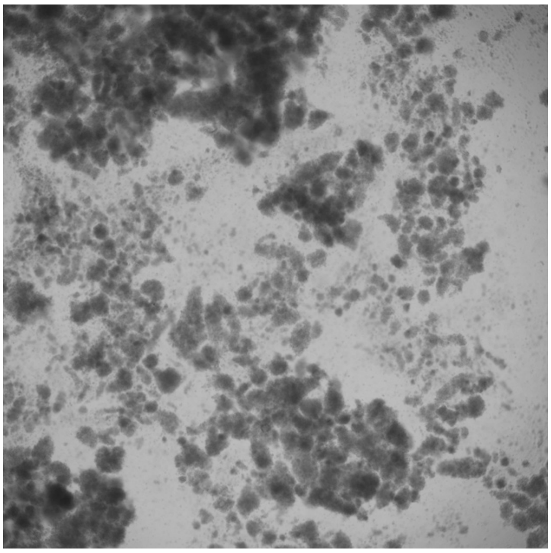

Supplementary Figure S7. Microscopic image showing cell aggregates formation in cultures of *P. kondratievae* as a response to growth on TSB + 5% NaCl for 24 hours at 42°C. A Nikon eclipse Ti2 (light) microscope was used with a 4X objective lens.

Supplementary Tables

Supplementary Table S1. Effect of C/N ratio on PHA production and biomass (CDW). Graphical representation of the data is shown in Figure 4. *only a single measurement was performed, all other measurements were performed in triplicates.

Carbon source	C/N	CDW (g/L)	PHA (g/L)	PHA [% of CDW]
Sodium gluconate	10	2.194 ± 0.078	0.704 ± 0.065	32.023 ± 2.134
	20	1.869 ± 0.103	0.440 ± 0.021	23.559 ± 0.660
	30	2.030 ± 0.015	0.486 ± 0.073	23.826 ± 1.885
	40	2.000 ± 0.210	0.423 ± 0.022	21.302 ± 1.380
	50	2.717 ± 0.127	0.442 ± 0.020	16.305 ± 1.229
Glucose	10	0.823 ± 0.017	0.151 ± 0.021	18.296 ± 2.072
	20	0.922 ± 0.070	0.170 ± 0.041	18.481 ± 4.495
	30	1.073 ± 0.079	0.340 ± 0.017	32.017 ± 3.931
	40	1.128 ± 0.030	0.401 ± 0.012	35.609 ± 1.512
	50	1.152 ± 0.090	0.290 ± 0.011	25.298 ± 1.187
Glycerol	10	1.508 ± 0.023	0.706 ± 0.020	46.824 ± 1.960
	20	1.35 ± 0.072	0.420 ± 0.031	30,987 ± 0.724
	30	1.367 ± 0.050	0.463 ± 0.052	33.785 ± 2.823
	40	1.156 ± 0.027	0.0214*	18.120*
	50	1.211 ± 0.061	0.278 ± 0.099	22.623 ± 7.479
